# The significance of elevated tumor markers among patients with interstitial lung diseases

**DOI:** 10.1038/s41598-022-20683-w

**Published:** 2022-10-06

**Authors:** Byoung Soo Kwon, Eun Sun Kim, Sung Yoon Lim, Myung Jin Song, Yeon Wook Kim, Hyung-Jun Kim, Yeon Joo Lee, Jong Sun Park, Young-Jae Cho, Ho Il Yoon, Choon-Taek Lee, Jae Ho Lee

**Affiliations:** 1 Division of Pulmonary and Critical Care Medicine, Department of Internal Medicine, Seoul National University Bundang Hospital, 82 Gumi-ro 173 Beon-gil, Bundang-gu, Seongnam-si, 13620 Gyeonggi-do Korea; 2 Department of Internal Medicine, Seoul National University College of Medicine, Seoul, Korea

**Keywords:** Respiratory tract diseases, Prognostic markers

## Abstract

The clinical implication of using serum tumor markers in patients with interstitial lung disease (ILD) is inconclusive. In this retrospective study, we analyzed the data of 1176 subjects (294 with ILDs and 882 healthy controls). Eligible patients were who had at least one or more available tumor marker results [carbohydrate antigen (CA) 19-9, CA 125, and carcinoembryonic antigen (CEA)] with no evidence of malignancies or other benign diseases that could be related to the increasing concentration of the values. The healthy controls selected from a health screening program were also screened for the presence of active cancer, and matched at a ratio of 1:3 with age and sex. The proportion of patients with abnormal values in the ILD group (121, idiopathic pulmonary fibrosis (IPF); 173, non-IPF-ILDs) was higher than in the matched control group (CEA, 21.5% vs. 5.5%; CA 19-9, 27.9% vs. 4.0%; CA 125, 36.4% vs. 2.0%). In the multivariable analysis, higher CEA levels were associated with shorter survival after adjusting for age, sex, lung function, and ILD subtypes (hazard ratio: 2.323, 95% confidence interval: 1.271–4.248, *P* = 0.006). In subgroup analysis, CEA remained a prognostic factor in patients with non-IPF-ILDs, but not in those with IPF.

## Introduction

Interstitial lung diseases (ILDs) encompass numerous types of disorders characterized by inflammation and/or fibrosis in the lung interstitium^1^. Idiopathic pulmonary fibrosis (IPF) is a major subset of idiopathic ILDs and causes significant mortality and morbidities^2^. The clinical trajectory of patients with IPF is highly variable, but advanced IPF is expected to have poor survival of 2–3 years after diagnosis^3^. Further, approximately one-third of non-IPF-ILDs showed a progressive phenotype similar to IPF^4–6^. High-resolution computed tomography (HRCT) scans and pulmonary function tests (PFT) are an important part of monitoring the disease^7,8^. However, the costs, radiation hazards, and suboptimal PFT results due to impaired lung functions, limit its use and necessitate other more easily measured prediction modalities. As a result, serum tumor markers have been proposed as a potential tool for predicting and monitoring patients at risk of disease progression or death.

To date, tumor markers are widely used for cancer screening programs, including colorectal, ovarian, and lung cancers, and cancers of the pancreato-biliary systems, etc^9–12^. However, most tumor markers are not ‘tumor-specific’. Rather, they are ‘tumor-associated’^13^; their serum concentrations originate from the proliferated epithelial cells regardless of the specific organ or system^14,15^. In addition, these biomarkers may be elevated in benign diseases^16–18^. Similarly, patients with chronic lung diseases without any evidence of malignancies may also show abnormal levels of tumor marker compared to the healthy population^18,19^. Additionally, previous studies have demonstrated that these values are associated with pulmonary functions^20,21^, disease progression, and increased mortality in patients with IPF^22^.

Despite the previous studies, no consensus has been established for the use of tumor markers. Furthermore, the relevance of tumor markers in patients with IPF and non-IPF-ILDs has yet to be determined. Thus, in the current study, we first investigated the differences in tumor marker concentrations between patients with ILDs and healthy populations. Then, we examined the prognostic roles of tumor markers in patients with various ILDs.

## Results

### Baseline characteristics

**Figure 1:**
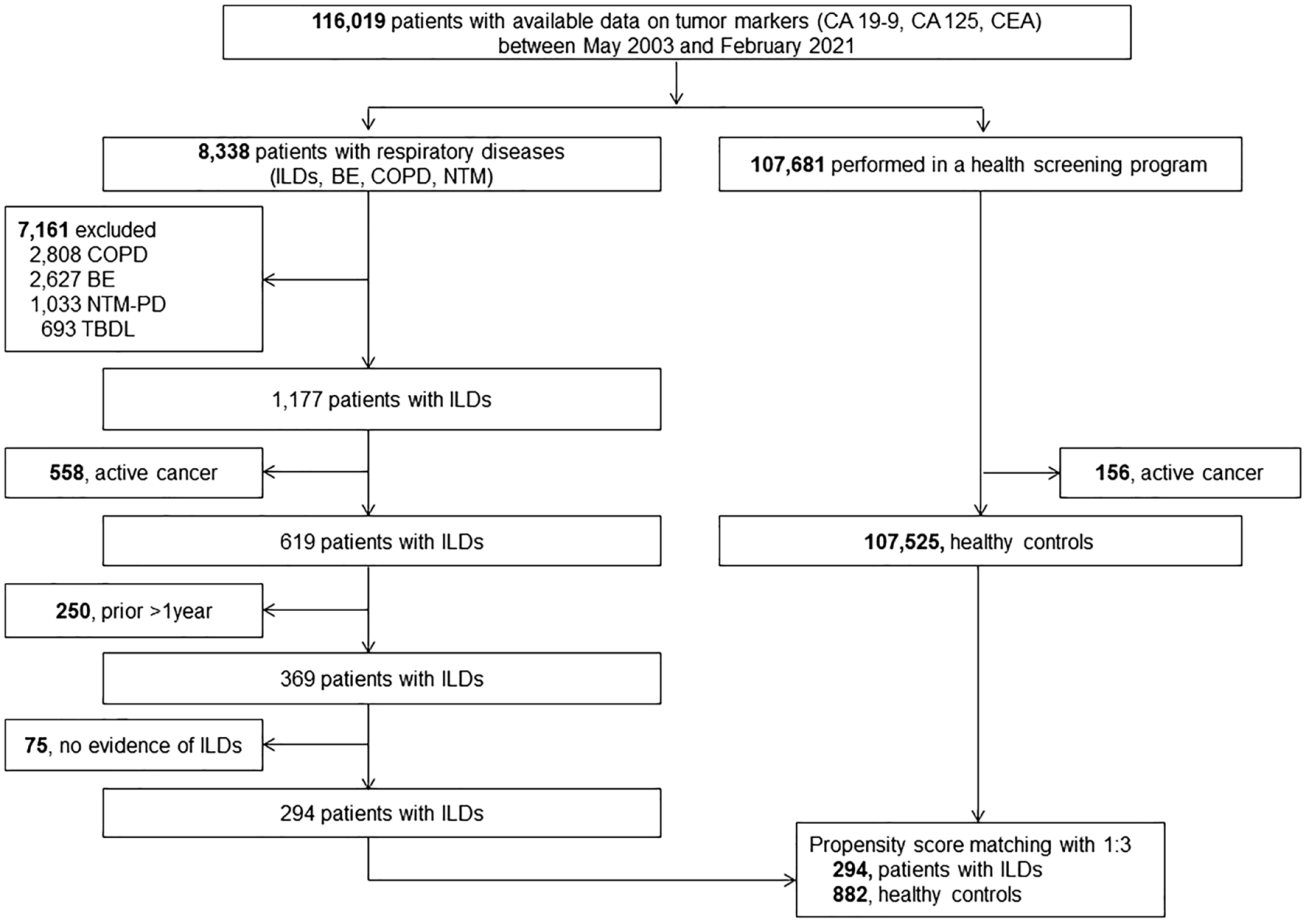
Study flow**.** *CA* carbohydrate antigen**,** *CEA* carcinoembryonic antigen, *ILD* interstitial lung disease, *BE* bronchiectasis, *COPD* chronic obstructive pulmonary disease, *NTM* nontuberculous mycobacterium, *TBDL *tuberculous destroyed lung.

In the unmatched cohort, healthy controls were more likely to be female and were younger than the ILDs group. After propensity score matching, a total of 294 patients with ILDs and 882 individuals in the healthy population were identified (Fig. 1). The mean age of all subjects was 68.1 years, and 66.4% were men (Table 1).

**Table 1:** Comparison of baseline characteristics between patients with ILDs and healthy controls.

	Unmatched		*P*-value	Matched		*P*-value
	ILDs (n = 294)	Control (n = 107,525)		ILD (n = 294)	Control (n = 882)	
Age	68.2 ± 12.9	48.0 ± 11.8	< 0.001	68.2 ± 12.9	68.1 ± 12.7	0.834
Sex, male (%)	192 (65.3)	57,311 (53.3)	< 0.001	192 (65.3)	589 (66.8)	0.643
CEA (ng/ml)	2.8 (1.7–4.9)	1.5 (1.0–2.3)	< 0.001	2.8 (1.7–4.9)	2.1 (1.4–3.0)	< 0.001
CEA (%)^a^	65 (24.4)	2252 (2.2)	< 0.001	65 (24.4)	48 (5.5)	< 0.001
CA19-9 (U/ml)	14.1 (5.9–34.8)	7.0 (4.4–11.7)	< 0.001	14.1 (5.9–34.8)	9.0 (5.8–13.9)	< 0.001
CA 19-9 (%)^a^	37 (24.0)	1011 (1.0)	< 0.001	37 (24.0)	35 (4.0)	< 0.001
CA125 (U/ml)	24.2 (11.2–59.4)	11.8 (8.7–16.6)	< 0.001	24.2 (11.2–59.4)	9.9 (7.6–14.1)	< 0.001
CA 125 (%)^a^	20 (36.4)	1902 (3.8)	< 0.001	20 (36.4)	6 (2.0)	< 0.001

Data are expressed as mean ± standard deviation (SD), median (interquartile range), or frequency (%).

The number of patients with available CEA, CA 19-9, and CA 125 results in matched cohort were 1143, 1034, and 348, respectively.

*ILD* interstitial lung disease, *CEA* carcinoembryonic antigen, *CA* carbohydrate antigen.

^a^Proportion of abnormal tumor marker values (normal range: CA 19-9, 0–37 U/mL; CA 125, 0–35 U/mL; CEA, 0–5 ng/mL).

The number of patients with available CEA, CA 19-9, and CA 125 results were 1143, 1034, and 348, respectively. Although the levels of tumor markers of the ILDs group were significantly higher than the matched control group, the median values of both groups were within normal limits. When the tumor markers were analyzed as dichotomized variables, a significantly higher proportion of patients in the ILD group showed abnormal values compared to individuals in the control group (Table 1).

Among the patients with ILDs, 121 patients (41.2%) were diagnosed with IPF, and the remaining 173 patients (58.8%) had non-IPF-ILDs. The IPF group was more likely to be older, male predominant, and had a higher proportion of ever-smoking history. There were no differences in the serum tumor marker levels between the groups in both continuous and categorical variables (Table 2).

**Table 2:** Baseline characteristics of 294 patients according to the diagnosis.

	Total (n = 294)	IPF (n = 121)	Non-IPF-ILDs (n = 173)^a^	*P*-value
Age	68.2 ± 12.9	72.5 ± 10.0	65.2 ± 13.9	< 0.001
Sex, male (%)	192 (65.3)	90 (74.4)	102 (59.0)	0.006
Smoking	162 (58.1)	79 (68.1)	83 (50.9)	0.004
**PFT**				
FVC	83.7 ± 28.4	81.1 ± 18.2	80.8 ± 19.7	0.899
DL_CO_	74.1 ± 23.6	78.7 ± 21.1	72.4 ± 24.4	0.089
**Tumor marker**				
CEA (n = 266)	2.8 (1.7–4.9)	3.0 (2.2–4.8)	2.7 (1.5–5.1)	0.236
CEA (%)^b^	65 (24.4)	24 (22.2)	41 (25.9)	0.487
CA19-9 (n = 154)	14.1 (5.9–34.8)	17.3 (6.3–80.3)	11.3 (5.7–31.0)	0.166
CA19-9 (%)^b^	37 (24.0)	16 (26.7)	21 (22.3)	0.556
CA125 (n = 55)	24.2 (11.2–59.4)	26.5 (17.4–65.0)	21.3 (10.7–60.1)	0.405
CA125 (%)^b^	20 (36.4)	6 (46.2)	14 (33.3)	0.401

Data are expressed as mean ± SD, median (interquartile range), or frequency (%).

*IPF* idiopathic pulmonary fibrosis, *ILD* interstitial lung disease, *PFT* pulmonary function test, *FVC* forced vital capacity, *DL*_*CO*_ diffusing capacity of the lung for carbon monoxide, *ILD* interstitial lung disease, *CEA* carcinoembryonic antigen, *CA* carbohydrate antigen.

^a^Non-IPF-ILDs: idiopathic nonspecific interstitial pneumonia (n = 16), cryptogenic organizing pneumonia (n = 11), connective tissue disease associated ILDs (n = 51), hypersensitivity pneumonitis (n = 1), unclassifiable ILD (n = 70), others (n = 24).

^b^Proportion of abnormal tumor marker values (normal range: CA 19-9, 0–37 U/mL; CA 125, 0–35 U/mL; CEA, 0–5 ng/mL).

### Survival and prognostic factors

**Figure 2:**
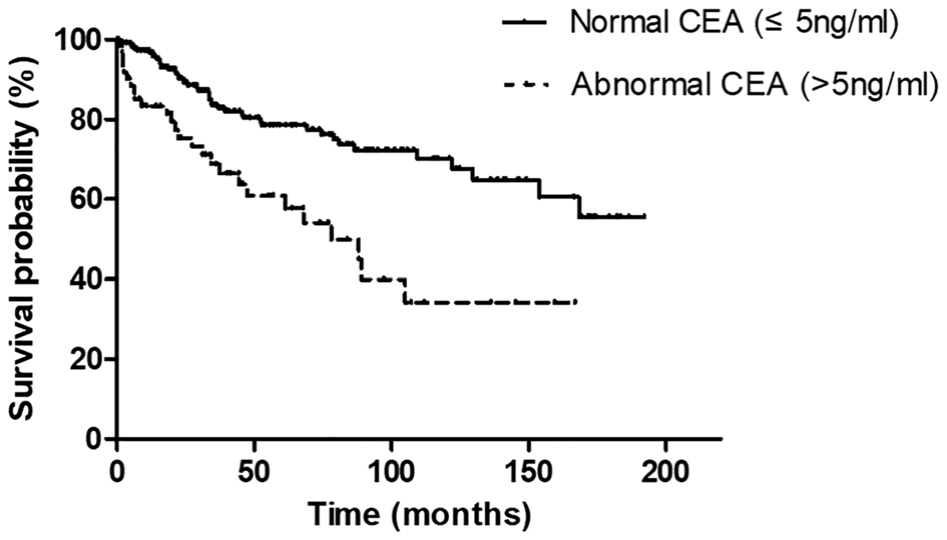
Comparison of survival in 294 patients with ILDs according to the serum carcinoembryonic antigen level. *CEA* carcinoembryonic antigen.

The median follow-up duration of the ILD group was 34.4 months, and 73 (24.8%) patients died. Patients with a high CEA concentration showed poorer survival than those with normal values (median survival of 78.0 months vs. not reached, *P* < 0.001, Fig. 2).

In univariate analysis, lower forced vital capacity (FVC), diagnosis of IPF, and higher values of tumor markers (CEA, CA 19-9, and CA 125) were significantly associated with mortality. In the multivariate analysis, we chose only CEA among the tumor markers because of missing CA 19-9 and CA 125 data. Consequently, a higher CEA level was significantly associated with mortality in patients with ILDs [hazard ratio (HR) 2.323, 95% confidence interval (CI) 1.271–4.248, *P* = 0.006] after adjusting for age, sex, FVC, and ILD types (Table 3).

**Table 3:** Prognostic factor for mortality in 294 patients with ILDs.

	Univariate analysis			Multivariate analysis		
	HR	95% CI	*P*-value	HR	95% CI	*P*-value
Age	1.010	0.991–1.028	0.300	–	–	–
Sex, male	1.356	0.822–2.237	0.233	–	–	–
Smoking	1.254	0.780–2.015	0.351			
**PFT**						
FVC (%)	0.963	0.949–0.978	< 0.001	0.959	0.944–0.975	< 0.001
DL_CO_ (%)	0.990	0.976–1.004	0.149			
ILD type, IPF	1.754	1.101–2.796	0.018	2.235	1.230–4.063	0.008
**Tumor markers**						
CEA	2.482	1.522–4.049	< 0.001	2.323	1.271–4.248	0.006
CA 19-9	2.540	1.230–5.247	0.012			
CA 125	6.624	1.238–35.446	0.027			

*HR* hazard ratio, *CI* confidence interval, *IPF* idiopathic pulmonary fibrosis, *ILD* interstitial lung disease, *PFT* pulmonary function test, *FVC* forced vital capacity, *DL*_*CO*_ diffusing capacity of the lung for carbon monoxide, *ILD* interstitial lung disease, *CEA* carcinoembryonic antigen, *CA* carbohydrate antigen.

In subgroup analysis according to the ILD subtypes, lower FVC was related to mortality in patients with IPF (HR 0.947, 95% CI 0.926–0.969, *P* < 0.001). In contrast, patients with lower FVC showed a trend toward shorter survival; however, this trend did not have statistical significance in non-IPF-ILDs. Instead, higher CEA was associated with poorer survival in patients with non-IPF-ILDs (HR 3.938, 95% CI 1.707–9.084, *P* = 0.001, Table 4).

**Table 4:** Prognostic factor for mortality according to the specific diagnosis.

	IPF			Non-IPF-ILDs		
	HR	95% CI	*P*-value	HR	95% CI	*P*-value
**Univariate analysis**						
Age	0.999	0.969–1.030	0.952	1.005	0.979–1.031	0.713
Sex, male	1.030	0.505–2.100	0.935	1.321	0.640–2.726	0.452
FVC (%)	0.949	0.929–0.969	< 0.001	0.972	0.952–0.993	0.009
CEA	1.362	0.658–2.818	0.405	4.621	2.243–9.520	< 0.001
**Multivariate analysis**						
FVC (%)	0.947	0.926–0.969	< 0.001	0.978	0.956–1.001	0.056
CEA				3.938	1.707–9.084	0.001

*HR* hazard ratio, *CI* confidence interval, *IPF* idiopathic pulmonary fibrosis, *ILD* interstitial lung disease, *PFT* pulmonary function test, *FVC* forced vital capacity, *ILD* interstitial lung disease, *CEA* carcinoembryonic antigen.

## Discussion

In this study, we compared the serum concentration of tumor markers between patients with ILDs and healthy subjects, and evaluated the prognostic role of these values, particularly in patients with ILDs. After matching with age and sex, all tumor marker levels were significantly higher in ILD groups than in healthy controls. Further, a higher level of CEA was associated with mortality in patients with ILDs, even after adjusting for the baseline demographics, ILD subtypes, and lung functions.

Currently, tumor markers are widely used in cancer screening programs. However, their diagnostic performance for cancer screening is limited^23^. Instead, tumor markers are used as a tool to monitor treatment response or tumor recurrence^24^. Moreover, because CA 19-9, CA 125, and CEA are synthesized in epithelial cells of various tissues, elevated tumor markers can be observed in nonmalignant diseases^16,18,25^. Chung et al. conducted a retrospective study using data of 25,786 subjects with health check-ups, and reported elevated CEA in 585 (2.3%) participants without malignancy compared to 12 (0.1%) participants with malignancy^26^. Associated conditions of COPD, smoking, or pulmonary inflammations were identified in 61.8% of the non-cancer subjects with elevated CEA. In a retrospective study, Hao et al. compared the CEA levels in patients with a benign or malignant disease with healthy controls^27^. The results showed that serum CEA concentrations were high even with no evidence of malignancy. CA 125, which is used for the detection of ovarian cancer, can also be elevated in benign diseases. In a population-based cohort study of 50,780 women in the United Kingdom, Funston et al. reported that 1,321 (2.6%) subjects with elevated CA 125 were categorized with non-ovarian causes^19^. Among them, 127 (9.6%) patients had associated respiratory causes. Similarly, Lee et al. investigated the positive rate of CA 19-9. Of a total of 58,498 subjects, 581 (1.0%) had elevated CA 19-9, and only four patients were diagnosed with cancer^28^. In our study, the proportion of subjects in the unmatched cohort without cancer and with elevated tumor markers was similar to the aforementioned studies. Although these proportions tended to increase after the propensity matching with age and sex, the tumor markers of patients with ILDs were significantly higher than those of the control group.

Many previous studies have assessed the association between tumor markers and clinical outcomes in patients with ILDs. Maher et al. retrospectively analyzed a prospective cohort of 312 patients with IPF and reported that baseline CA 19-9 and changes in CA 125 were potential biomarkers for disease progression and overall survival, respectively^22^. Dai et al. found that CA 19-9, CA 125, and CEA were higher in patients with IPF than in patients with other chronic respiratory diseases^29^. Similarly, tumor markers were also elevated in patients with non-IPF-ILD^30–33^. However, even with emerging research, the results are inconclusive, and the question of which biomarkers give the most convincing evidence should be further elucidated. In our study, we found that all tumor markers were significantly higher in the ILD group than in the controls. Further, the proportion of patients with elevations greater than the upper limit of tumor markers was 21.5–36.4%. Nevertheless, there were no differences between patients with IPF and non-IPF-ILDs. According to previous studies, the levels of tumor markers that originated from epithelial cells reflect the severity of diseases^21^. Thus, tumor markers, especially CEA, were negatively correlated with lung functions^20,21^. Taken together, the similar tumor marker levels in the IPF and non-IPF-ILDs groups noted in our study might be because there were no differences in lung function between the two groups.

We focused on the clinical relevance of CEA in the multivariable analysis because of missing CA 19-9 and CA 125 data. In the results, higher CEA levels showed poorer survival in patients with ILDs as a whole, even after adjusting for confounders. Evidence has shown that distal airways and type II alveolar cells play a critical role in the pathogenesis of lung fibrosis^34–36^. Fahim et al. demonstrated that strong CEA staining was present in the epithelial cell lining of the respiratory bronchioles and the honeycomb cysts of lung tissue in patients with IPF^21^. Therefore, although the mechanism of CEA elevation in ILDs is still unclear, it could be postulated that the serum concentration of CEA is linked to the severity of fibrosis^27,31,32^. However, in the subgroup analysis of our study, the IPF group did not show sustained results. A possible explanation for this discrepancy is because of the small sample size of the IPF group.

There are several limitations in our study. First, data on the smoking status of unmatched healthy controls were not collected. It is known that serum CEA concentrations are increased in heavy smokers^37^. Nevertheless, the tumor markers in the control group were similar to the previous population-based studies. In addition, smoking status between the two groups were not significantly different (51% in healthy controls vs. 58.1% in ILDs, *P* = 0.052, data not shown). Second, dynamic changes of tumor markers were not evaluated in the current study. For instance, Maher et al. reported that the serial rising concentration of CA 125 is a prognostic marker in patients with IPF^22^. Although our study did not provide information on the association between serial tumor marker changes and clinical outcomes, the results give us useful information for predicting long term prognosis at the time of patient diagnosis. This is possible because we used blood test results taken as close as possible to the date of the diagnosis of ILD. Third, other potential serum biomarkers for predicting clinical courses of patients with ILDs such as Krebs von den Lungen-6 or serum surfactant protein, were not assessed in the current study. Fourth, the number of patients with specific disease entities of non-IPF ILDs was relatively small to draw robust conclusions. Moreover, because of the heterogeneity of it nature, these patients might show a different pattern of association between serum CEA levels and clinical outcomes. Nevertheless, the CEA levels at the time of diagnosis were found to be significant even after adjusting for the clinical relevant factors. Considering the simplicity of measurement, CEA could be a useful biomarker for identifying poor prognostic subgroups.

In conclusion, the serum levels of CEA, CA 19-9, and CA 125 of patients with ILDs were significantly higher than the age- and sex-matched healthy subjects. In addition, baseline CEA at ILD diagnosis showed potential as a predictor of prognosis, particularly in patients with non-IPF-ILDs. Further research with sufficient sample size is required in order to verify these results for patients with each specific disease.

## Methods

### Study population

In this retrospective study, we reviewed 8338 patients diagnosed with respiratory diseases and 107,681 subjects who underwent a health screening program between May 2003 and February 2021. Subjects with at least one or more available data on tumor markers [carbohydrate antigen (CA) 19-9, CA 125, and carcinoembryonic antigen (CEA)] were eligible for the study. To minimize the effect of chronic respiratory diseases on tumor markers, we excluded patients with chronic obstructive pulmonary disease (COPD), bronchiectasis, nontuberculous mycobacterial pulmonary disease, and tuberculosis-destroyed lung. In addition, patients with active cancer who were treated with anticancer therapy or those with shorter than one-year interval between the date of ILD diagnosis and date of cancer diagnosis were excluded. Similarly, patients without malignant diseases but with a date of tumor marker evaluation beyond 1-year before or after ILD diagnosis were also excluded. IPF was diagnosed based on the criteria of the 2018 American Thoracic Society (ATS)/European Respiratory Society (ERS)/Japanese Respiratory Society (JRS), and Latin American Thoracic Society (ALAT) statement^38^. Patients with ILDs who did not fulfill the ATS/ERS/JRS/ALAT diagnostic criteria were categorized as non-IPF-ILD.

The tumor markers of eligible patients were compared with those of healthy controls without active cancers using propensity score matching at a ratio of 1:3 with age and sex.

The study was approved by the Institutional Review Board (IRB) of Seoul National University of Bundang Hospital (IRB No. B-2012-654-004) and is consistent with the principles of the Declaration of Helsinki. Informed consent was waived by the IRB of Seoul National University of Bundang Hospital due to the retrospective nature of the study.

### Measurement of serum tumor markers

Serum concentrations of CA 19-9, CA 125, and CEA were measured by electrochiluminescence immunoassay or immunoradio metric assay. The reference values were in the range 0–37 U/ml for CA 19-9, 0–35 U/ml for CA 125, and 0–5 ng/ml for CEA.

### Statistical analysis

Continuous variables were expressed as mean ± standard deviation (SD) or median (interquartile range, IQR), and analyzed by Student’s *t*-test. Categorical variables were presented as frequency and compared by the chi-squared test. The propensity score matching was performed by sex and age at the test date.

The overall survival was estimated using the Kaplan–Meier method. The survival time was calculated from the date of ILD diagnosis to the last hospital visit or time of death. Cox proportional hazards regression analysis was used to identify significant variables related to survival. Variables with a *P*-value < 0.1 in the unadjusted analysis and known as having clinical relevance were chosen in multivariable analysis using the backward log-likelihood ratio method. A *P*-value of < 0.05 was considered significant. Statistical analyses were performed using IBM SPSS version 25 (SPSS, Inc., Chicago IL, USA), R-studio, and Prism version 5 (GraphPad, San Diego, CA, USA).

## Data Availability

The data that support the findings of this study are not openly available due to the fact that consent to sharing data was not obtained from participants. However, the datasets used and analyzed in the current study are available from the corresponding author on reasonable request.
